# Transforming Growth Factor-β (TGF-β) Expression Is Increased in the Subsynovial Connective Tissue in a Rabbit Model of Carpal Tunnel Syndrome

**DOI:** 10.1371/journal.pone.0108312

**Published:** 2014-09-30

**Authors:** Takako Chikenji, Anne Gingery, Chunfeng Zhao, Matthias Vanhees, Tamami Moriya, Ramona Reisdorf, Kai-Nan An, Peter C. Amadio

**Affiliations:** 1 Department of Orthopedic Surgery, Mayo Clinic, Rochester, Minnesota, United States of America; 2 Department of Biochemistry and Molecular Biology, Mayo Clinic, Rochester, Minnesota, United States of America; Institut national de la santé et de la recherche médicale - Institut Cochin, France

## Abstract

Carpal tunnel syndrome (CTS) is an idiopathic disease that results from increased fibrosis of the subsynovial connective tissue (SSCT). A recent study found overexpression of both transforming growth factor-β (TGF-β) and connective tissue growth factor (CTGF) in the SSCT of CTS patients. This study investigated TGF-β and CTGF expression in a rabbit model of CTS, in which SSCT fibrosis is induced by a surgical injury. Levels of TGF-β1 and CTGF at 6, 12, 24 weeks after injury were determined by immunohistochemistry A significant increase in TGF-β1 and a concomitant significant increase in CTGF were found at 6 weeks, in addition to higher cell density compared to normal (all p<0.05), Interestingly, CTGF expression was reduced at 12 and 24 weeks, suggesting that an initial insult results in a time limited response. We conclude that this rabbit model mimics the fibrosis found in human CTS, and may be useful to study pathogenetic mechanisms of CTS *in vivo*.

## Introduction

Idiopathic CTS [1], [2] is characterized as well by non-inflammatory fibrosis of the (SSCT) within the carpal tunnel. Although the relationship between the repetitive motion and the fibrosis is still unclear [3], [4], an increased expression of transforming growth factor-β (TGF-β) and connective tissue growth factor (CTGF) has recently been found in the SSCT of CTS patients [5]. TGF-β and CTGF are profibrotic cytokines, and have been found in other sites of non-inflammatory progressive fibrosis as well [6]–[10].

While the presence of TGF-β and a downstream cytokine, CTGF, suggests strongly that TGF-β signaling is implicated in SSCT fibrosis, clinical data from the SSCT of CTS patients is limited, due to the need for biopsy, which is most commonly accomplished at the time of surgery, when the CTS is well advanced. In order to truly understand the role of SSCT pathology in CTS, a time-dependent investigation is necessary, which in turn implies the need for a valid animal model.

Therefore, recently, a rabbit model for CTS has been developed to understand the pathomechanism of idiopathic CTS as previously described [11], [12]. Briefly the model is created by surgically stretching SSCT, thereby disrupting the normal elasticity of the tissue. Pathological alterations in this model include increased fibroblast proliferation and overexpression of TGF-β receptors [11]–[13], suggesting that the shearing injury activates TGF-β signaling.

The primary goal of this study was to evaluate expression of TGF-β1 and CTGF in the SSCT in this CTS animal model, and to test the hypothesis that, as in human CTS, TGF-β1 and CTGF are upregulated in this animal model of SSCT shear injury. If TGF-β1 and CTGF are upregulated in this animal model of shear injury, the results would support the hypothesis that shear injury to the SSCT might be a potential cause of CTS, as might occur in repetitive injury.

## Methods

### Ethics Statement

This study was carried out in strict accordance with the recommendations in the Guide for the Care and Use of Laboratory Animals of the National Institutes of Health. The Mayo Clinic Institutional Animal Care and Use Committee (IACUC) approved all animal care and experimental procedures described in this paper (Protocol A3291-01). Tissues were harvested after euthanasia following Mayo Clinic IACUC-approved protocols at the designated time, animals were anesthetized with an intramuscular injection of Ketamine (40 mg/kg) and Xylazine (6 mg/kg) and then sacrificed with an overdose of pentobarbital IV (100 mg/kg)All efforts were made to minimize suffering.

**Figure 1:**
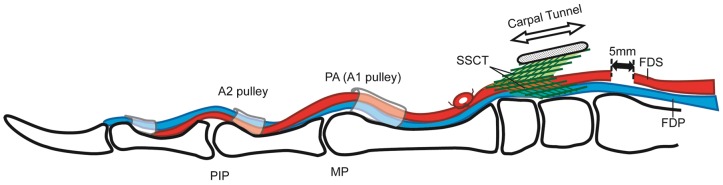
Schematic drawings of surgical procedure.

A total of 32 New Zealand White female rabbits, weighting 3.2–5.2 kg, were used in this study. The study was approved by our Institutional Animal Care and Use Committee. Rabbits were anesthetized with an IM injection of Ketamine (40 mg/kg), Xylazine (6 mg/kg), and Acepromazine (1 mg/kg) and maintained on Isoflurane (1.5–3%) continuously until the end of surgery. Buprenorphine SR (0.15 mg/kg) was given subcutaneously once before surgery for pain, and postoperatively as needed. SSCT fibrosis was created by a surgical intervention described previously [11], [12]. Briefly, two volar incisions were made, proximal and distal to the carpal tunnel, and the flexor digitorum superficialis (FDS) tendon of the middle digit was cut at the musculotendinous junction. The middle digit FDS tendon distal to the carpal tunnel was then shortened by 5 mm, thereby stretching the surrounding SSCT by that amount (Figure 1). After surgery, the rabbits were allowed full cage activity. Rabbits were housed singly in cages that were 27″×27″×18″ and were given water and pellet feed *Ad libitum*. The room was on a light and dark cycle of 12 hours with the temperature of the room being kept between 16° to 22°C. Eight untreated rabbits were used as normal controls.

### Immunohistochemistry

Immediately after sacrifice, formalin-fixed, paraffin embedded 4 µm sections of SSCT were prepared for hematoxylin and eosin (HE) and immunohistochemical (IHC) staining. Sections were then deparaffinized in xylene and rehydrated with graded ethanol for HE and IHC staining. The IHC staining was performed as described previously [5]. Primary antibodies for TGFβ1 (R&D, Minneapolis, MN), and CTGF (Santa Cruz Biotechnology, Santa Cruz, CA) were diluted 1∶200 and incubated for 12 hours in 0.1 M PBS with c 0.3% Triton X-100 (PBST) at 4C°. After washing for 30 minutes with 0.1 M PBST, secondary antibodies for TGFβ1 (Mouse IgG, Vector Laboratories, Burlingame, CA) and CTGF (Goat IgG, Vector Laboratories, Burlingame, CA) were incubated for 2 hours diluted 1∶500 in PBST at room temperature. These sections were then washed with PBST for 30 minutes and incubated for 2 hours in avidin-biotin-peroxidase complex (ABC kit, Vector Laboratories, Burlingame, CA) diluted 1∶1000 in PBST at room temperature. The immunoreaction was visualized by incubation in 0.05 M Tris-HCl buffer (pH 7.6) containing 0.01% 3,3'-diaminobenzidine (DAB) (Sigma-Aldrich, St Louis, MO), and 0.0003% H_2_O_2_ for 30 minutes at room temperature. Slides were counterstained with hematoxylin QS (Vector Laboratories) as per manufacturer protocols. Negative control slides were prepared by two methods, eliminating the primary antibody and replacing the primary antibody with species matched sera to the primary antibody. Three areas within the SSCT surrounding the middle digit FDS tendon were imaged digitally (400x). Cell density, TGF-β1 positive cells and CTGF positive cells were quantified in each image. A previous study has shown reliable intraclass correlation coefficients (ICCs) for intra-observer reliability for this method [5].

### Statistical Methods

Results were expressed as a mean ± standard deviation (SD). An effect size calculation was performed by a pilot study to detect differences in cell densities, TGF-β1 and CTGF between CTS animal models and controls. The effect size for cell densities was 1.15, TGF-β1 was 1.19, and CTGF was 0.64. A sample size of 4 for cell densities, of 4 for TGF-β1, and of 8 for CTGF were estimated from the effect size in each groups of 80% power at a significance level of p<0.05. One way ANOVA and the Tukey-Kramer test was used to compare TGF-β1 and CTGF positive cells between experimental and control animals. Correlation between TGF-β1 and CTGF was assessed using the Pearson correlation coefficient. Values of *p*<0.05 were considered to be statistically significant for all the statistical analyses. All analyses were performed with JMP 9 software (SAS Institute Inc., Cary, NC).

## Results

**Figure 2:**
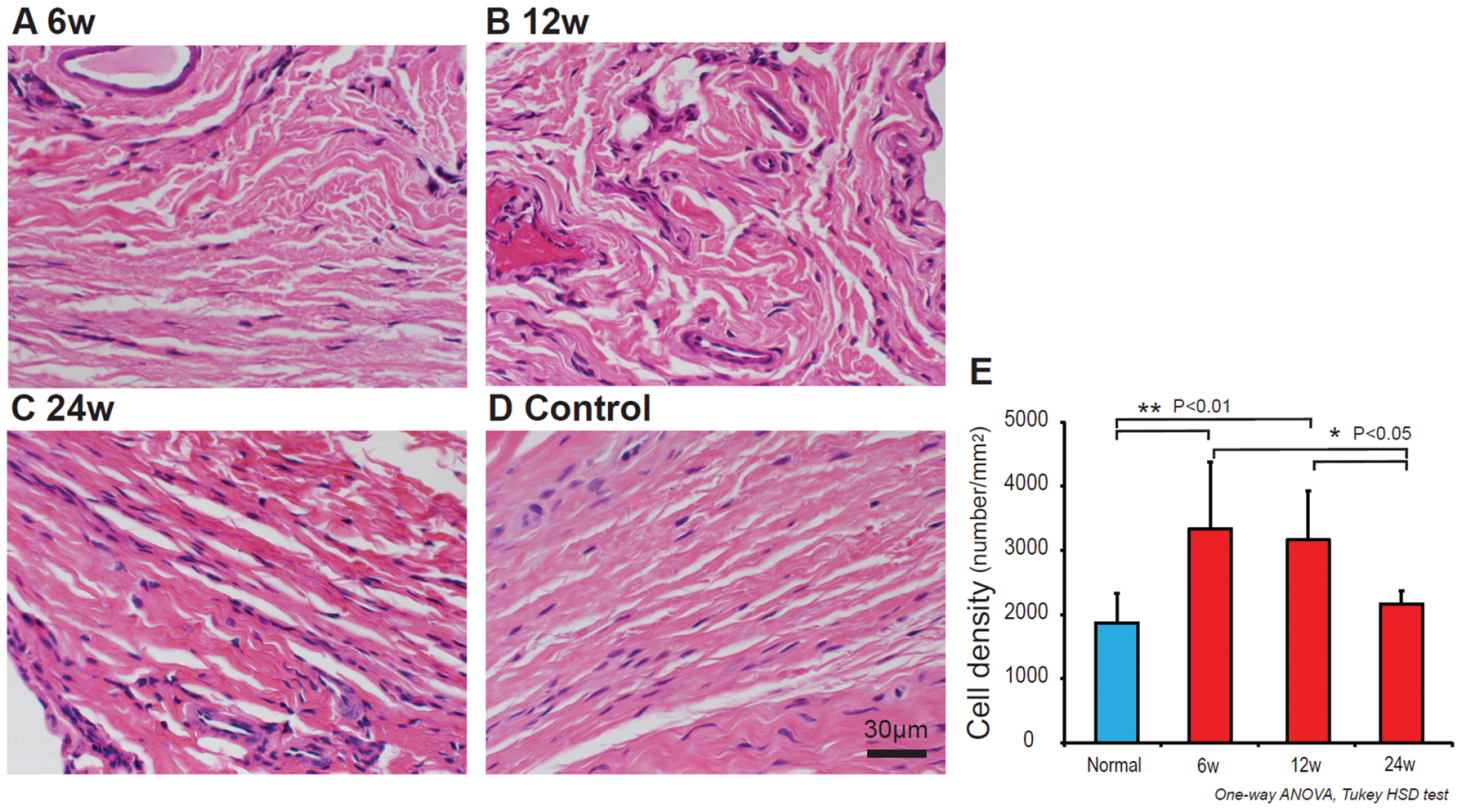
HE staining of 6 week (A), 12 week (B), 24 week (C) shear injury rabbit model and control (D). Cell density in the 6 and 12 weeks shear injury rabbit model was larger than that in the controls and 24 weeks shear injury rabbit model (E).

Figure 2 shows the HE staining in the 6 weeks (Figure 2A), 12 weeks (Figure 2B) and 24 weeks (Figure 2C) shear injury animals and in the control animals (Figure 2D). Cell densities in the 6, 12 and 24 weeks shear injury animals, and controls were 1871.5±460.1 mm^2^, 3334.9±1043.1 mm^2^, 3162.7±771.2 mm^2^, and 2168.20±203.7 mm^2^, respectively. The cell density in the 6 and 12 weeks shear injury animals was significantly larger than that in the controls (*p*<0.01) and 24 week shear injury animals (*p*<0.05) (Figure 2E).

**Figure 3:**
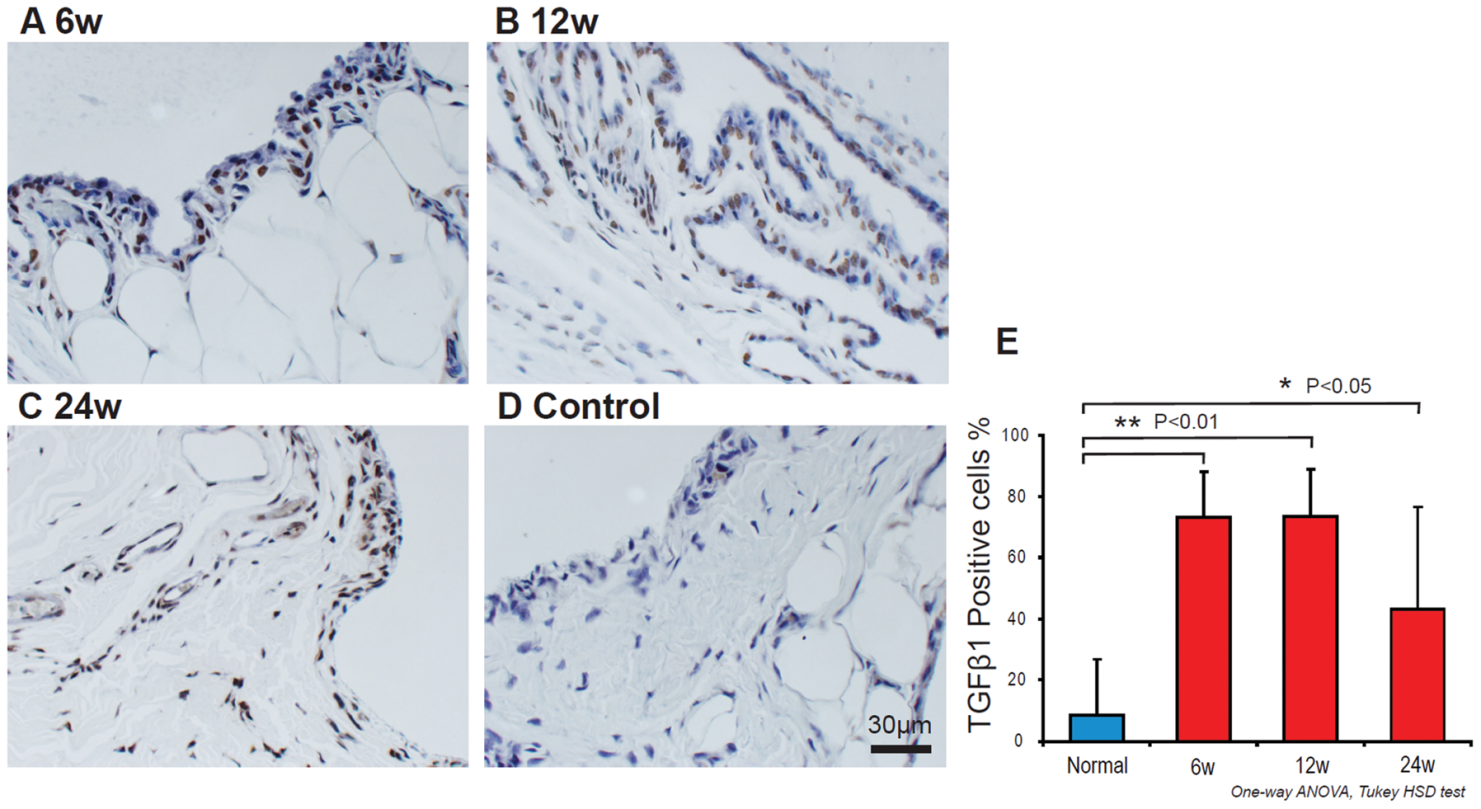
TGF-β1 expression of 6 week (A), 12 week (B), 24 week (C) shear injury rabbit model and control (D). The percentage of the TGF-β1 positive cells was increased in shear injury rabbit model compared to control (E).

Figure 3 shows the characteristic TGF-β1 staining of 6 week (Figure 3A), 12 week (Figure 3B), 24 week shear injury (Figure 3C) and control animals (Figure 3D). The percentage of the TGF-β1 positive cells was 73.4±14.9% at 6 week, 73.5±15.5% at 12 week, 43.3±33.6 at 24 weeks shear injury animals, and 8.6±18.2% in control animals. The TGF-β1 positive cells were increased in the shear injury animals at 6 weeks (*p*<0.01), 12 weeks (*p*<0.01) and 24 weeks (*p*<0.05) as compared to controls (Figure 3E).

**Figure 4:**
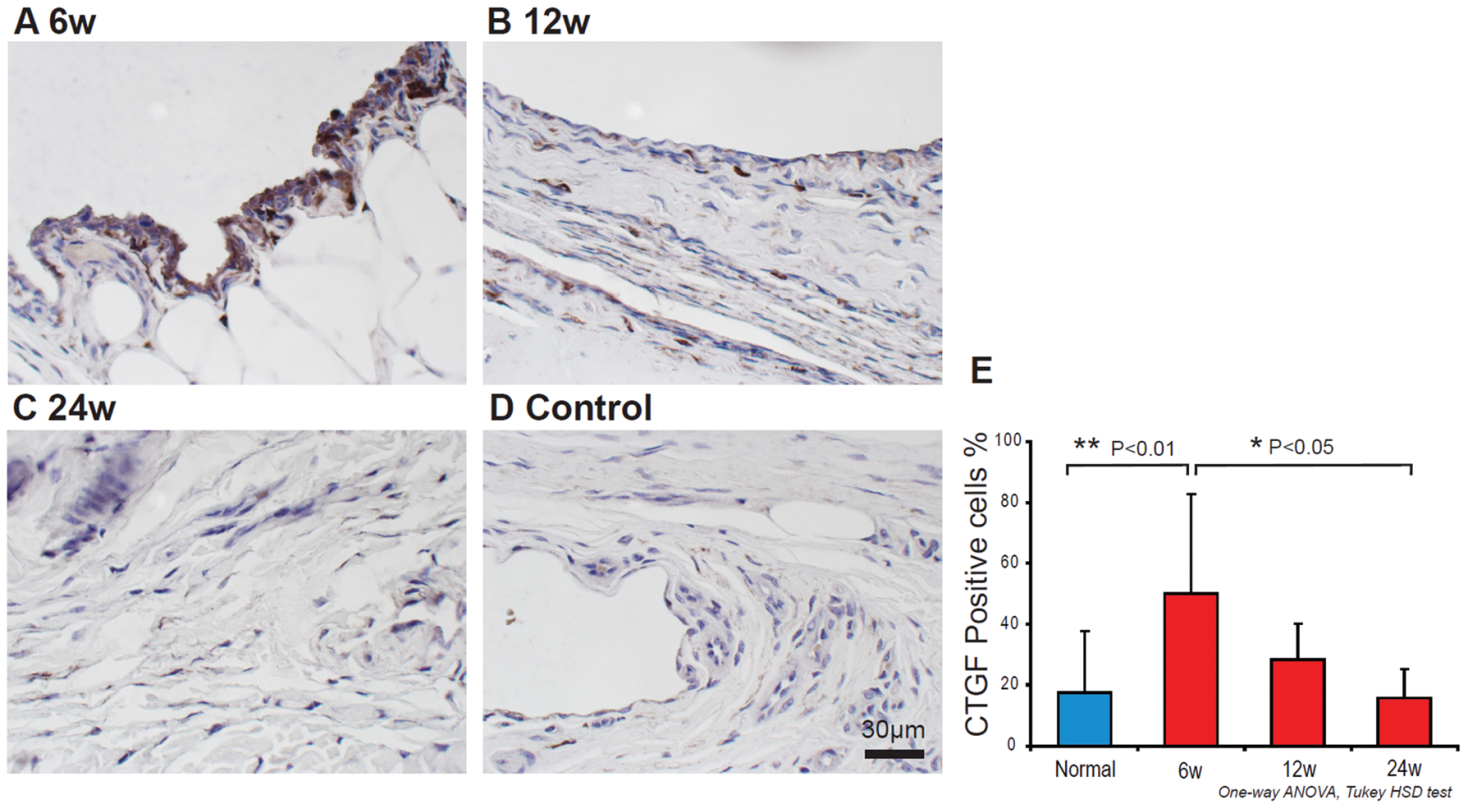
CTGF expression in 6 (A), 12 (B), 24 week (C) shear injury rabbit model and control (D). The percentage of the CTGF positive cells were increased in 6 week compared to control and 24 week shear injury rabbit model (E).

**Figure 5:**
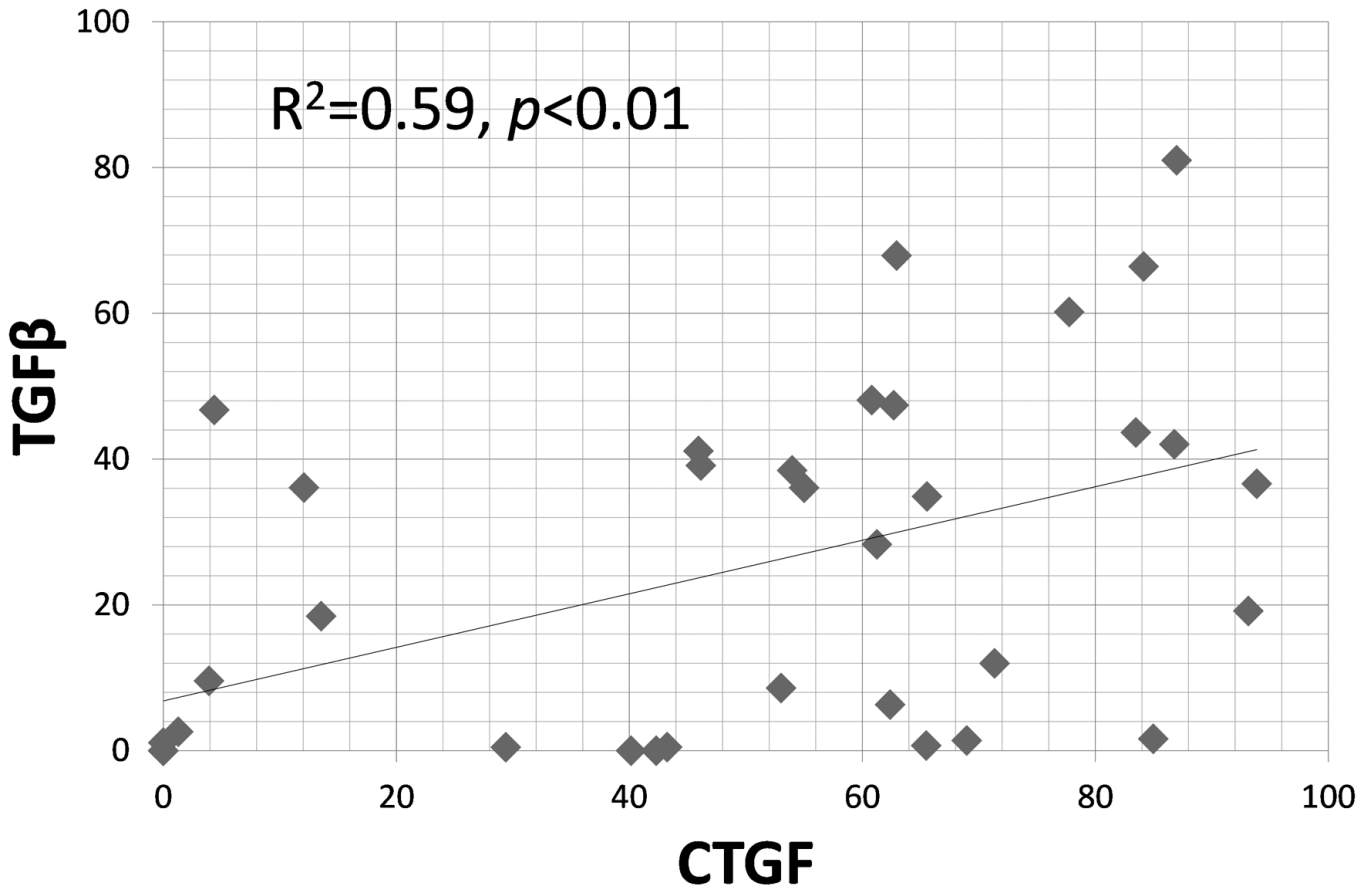
Pairwise correlation coefficient between TGF-β1 and CTGF. A moderate correlation was found between TGF-β1 and CTGF.

Positive CTGF cells were also found in 6 and 12 week shear injury animals (Figure 4A and 4B). However by 24 weeks there were fewer CTGF positive cells in the experimental animals (Figure 4C), and few CTGF positive cells were seen in the controls (Figure 4D). The percentage of CTGF positive cells was 50.0±32.5% in the 6 week, 28.3±11.9% in the 12 week, and 15.8±9.6 in the 24 week shear injury animals, and 17.6±20.0% in the control animals. The percentage of CTGF positive cells were significantly increased in the 6 week shear injury animals compared to control (*p*<0.05) and 24 week shear injury model animals (*p*<0.05) (Figure 4E). TGF-β1 and CTGF showed a moderate positive correlation (R^2^ = 0.59, *p*<0.01) (Figure 5).

## Discussion

In an animal model of SSCT fibrosis, we have demonstrated that TGF-β1 and CTGF are increased at 6 weeks, with higher cell density compared to normal. The overexpression of both TGF-β1 and CTGF in the SSCT of the shear injury rabbit model further supports the hypothesis that TGF-β signaling may account, in large part, for SSCT fibrosis in this animal model, as is seen in CTS patients [5].

Tissue fibrosis results from a dysregulation of wound healing [7], [14]. Immediately after injury, TGF-β is consistently present in wound fluid. [15], [16] The expression of TGF-β in wound fluid peaks at 7 to 14 days and returns to baseline levels at day 16 after injury in rat skin [17]. CTGF is also expressed in fibroblasts during the wound healing process [18]. The highest level of CTGF was observed at day 9 after injury, which corresponds with the early stages of granulation tissue in rat skin [18]. Generally, these growth factors are suppressed as the normal wound healing process continues [15]. However, repeated injury results in increased TGF-β1, leading to the continued production of extracellular matrix and tissue fibrosis [7]. Exposure to TGF-β or CTGF results in transient effects, whereas a chronic response is found when both growth factors are present [19], [20]. The expression of these growth factors in fibrotic SSCT implicates TGF-β and CTGF in the maintenance of chronic fibrosis.

In this study, a shear injury was created surgically in rabbit SSCT. At 6 weeks after injury, high expressions of TGF-β1 and CTGF were still observed, with increased cell density. These results support the hypothesis that shear injury to the SSCT might be a potential cause of CTS. Such an injury might occur with repetitive injury, which has indeed been proposed as a potential etiology of CTS [1], [2]. Recent studies have reported that TGF-β signaling and bioavailability results both from cellular and mechanical forces. Maeda et al. showed that mechanical forces regulate TGF-β and Smads, downstream mediators of TGF-β signaling, in tenocytes [21]. Another study showed that mechanical forces can increase TGF-β bioavailability by releasing TGF-β from TGF-β latency associated protein (LAP). This study determined that 40 pN force is required to disassociate LAP from TGF-β. This force can be generated from integrin linkages between cells and matrix [22]–[24]. Indeed matrix stiffness has been shown to increase tractions with corresponding matrix stiffness. Mechanical stress also increases the expression of CTGF. Miyake et al. [25] have reported that cyclic stretch increases the expression of CTGF in fibroblasts. Finally, mechanical stress has been shown to increase CTGF expression in three dimensional collagen matrices and release of this tension results in a concomitant and substantial decrease in CTGF [26], [27]. During pathological conditions this relationship is perturbed. TGF-β stimulates fibroblasts to deposit extracellular matrix, as well as down regulates matrix metalloproteases, such as collagenase. This leads to a vicious cycle of TGF-β mediated fibrotic signaling including increased matrix deposition [28]. In addition, previous work has shown that CTGF is induced during TGF-β signaling [29], [30].

In this study, TGF-β1 was overexpressed at 6, 12 and 24 weeks after a single shear injury to the SSCT, CTGF was overexpressed at 6 weeks, and both CTGF expression and fibroblast density returned to normal at 24 weeks. Furthermore, in our previous studies, electrophysiological evaluations were performed in the same rabbit model [11], [12]. The median nerve distal motor amplitude was decreased in 12 weeks after a single shear injury [11], but not in the 6 and 24 weeks [12]. In human CTS, both TGF-β1 and CTGF are overexpressed at all stages of CTS severity, or, at least, those stages in which surgery is performed, since that data comes from specimens collected at the time of carpal tunnel release surgery. These clinical findings, combined with our experimental ones, suggest that a shear injury to the SSCT could be a trigger of SSCT fibrosis, and that repeated injury or mechanical stress might boost the expression of the profibrotic growth factors, and create a vicious cycle of SSCT fibrosis, resulting eventually in human idiopathic CTS.

Limitations of this study include evaluating only TGF-β isoform 1. There are three isoforms, TGF-β1, 2 and 3. The isoforms have approximately 80% structural homology and redundant biological functions. Regarding fibrotic actions, TGF-β1 has been identified as the most prominent and bioactive isoform in various tissue fibrosis [7], [31]. However, a distinct function in each isoform has been reported in wound healing and fibrosis [6], [32]. In a cutaneous wound model, excessive production of TGF-β1 and 2 isoforms promotes scar formation, while an increase TGF-β3 relative to TGF-β1 and 2 reduces extracellular matrix accumulation such as type III collagen [33]. Although we have demonstrated that increased TGF-β1 in SSCT fibrosis of CTS patients with type III collagen [5], the balance of the three isoforms might be underlying mechanism of SSCT fibrosis. In addition, collagen synthesis, which is stimulated by the TGF-β1 and CTGF, might change over time after the shear injury. In the human SSCT of CTS patients, the synthesis of collagen was correlated with these growth factors [5]. Although we only noted profibrotic growth factors in this study, characteristic of extracellular matrix after a shear injury may provide further insight into the pathogenesis of CTS.

In this study we explored the CTS rabbit model out to 24 weeks after one shear injury. It should be noted that the temporal observations concerning the fibrotic growth factors after just one injury results in substantial profibrotic changes. In the chronic human condition of CTS this fibrosis would likely be sustained over time [34].

Recently, various inhibitors of TGF-β signaling have been developed in fibrosis and cancer treatments. Most of large molecular weight inhibitors, which affect the ligand of TGF-β, are monoclonal antibody that bind and inactivate all three isoforms [35], TGF-β1 [36], and TGF-β2 [37]. On the other hand, small molecular weight inhibitors are developed to inhibit the TGF-β receptor I kinase [38], and antisense oligonucleotide of TGF-β blocks production of TGF-β by targeting the mRNA of TGF-β for degradation [39]. Future studies will evaluate the efficacy of targeted therapies to disrupt the SSCT fibrosis in this animal model.
